# Present and past selves: a steady-state visual evoked potentials approach to self-face processing

**DOI:** 10.1038/s41598-017-16679-6

**Published:** 2017-11-27

**Authors:** I. Kotlewska, M. J. Wójcik, M. M. Nowicka, K. Marczak, A. Nowicka

**Affiliations:** 1 Laboratory of Psychophysiology, Department of Neurophysiology, Nencki Institute of Experimental Biology, 3 Pasteur St., 02-093 Warsaw, Poland; 2 Wroclaw Faculty of Psychology, SWPS University of Social Sciences and Humanities, 30b Ostrowskiego St., 53-238 Wroclaw, Poland; 3 Faculty of Physics, Warsaw University of Technology, 75 Koszykowa St., 00-662 Warsaw, Poland

## Abstract

The self-face has a prioritized status in the processing of incoming visual inputs. As the self-face changes over the lifespan, this stimulus seems to be well-suited for investigation of the self across time. Here, steady-state visual evoked potentials (SSVEP, oscillatory responses to periodic stimulation with a frequency that mirrors the frequency of stimulation) were used to investigate this topic. Different types of faces (present self, past self, close-other’s, unknown, scrambled) flickered four times per second in two types of stimulation (‘identical’, with the same image of a given type of face; ‘different’, with different images of the same type of face). Each of the 10 stimulation sessions lasted 90 seconds and was repeated three times. EEG data were recorded and analyzed in 20 participants. In general, faces evoked higher SSVEP than scrambled faces. The impact of identical and different stimulation was similar for faces and scrambled faces: SSVEP to different stimuli (faces, scrambled faces) was enhanced in comparison to identical ones. Present self-faces evoked higher SSVEP responses than past self-faces in the different stimulation condition only. Thus, our results showed that the physical aspects of the present and past selves are differentiated on the neural level in the absence of an overt behavior.

## Introduction

The self is an extensively investigated topic in both cognitive neuroscience and psychology despite the fact that this term is difficult to define and is often used to discuss multiple different cognitive phenomena. Some of the most prominent and influential philosophers and psychologists have theorized about the self. More than a century ago William James^1^ noted that the self is not a single primordial entity. This early conceptualization set the stage for later work focusing on multiple facets of the self.

Gillihan and Farah^2^ proposed a distinction between *psychological* and *physical* aspects of the self; while psychological aspects of the self tend to be operationalized with studies examining autobiographical memory and self-knowledge, physical aspects of the self are typically examined in studies of self-face recognition. Gallagher^3^, in turn, delineates yet another distinction: ‘minimal’ self vs. ‘narrative’ self. In his view, the ‘minimal’ self is referred to as the self devoid of temporal extension; (phenomenologically, a consciousness of oneself as an immediate subject of experience), whereas the ‘narrative’ self involves personal identity and the sense of self-continuity across time that is based on the ability to consolidate different and temporally separated pieces of self-related information into a one coherent whole^4,5^.

The self continually evolves as time passes, and the self-concept is updated in order to account for these alterations^6,7^. Time-related changes of physical aspects of the self refer, for instance, to the processes of aging, weight gain/loss, etc. Such changes exert an influence on the appearance of the self-face. Thus, one’s own present and past faces seem to be appropriate to study the neural correlates of time-related changes in the physical self.

However, only a few event-related potential (ERP) studies have investigated the self-continuity across time with the usage of present and past faces^8,9^. The results of those studies revealed that – on the neural level – differences between the present and past physical self were absent when images of the present self-face were compared to images of the self-face in adolescence and early adulthood^8,9^. It is worth noting that detection^8^ or identification^9^ of faces was required in the aforementioned studies. This raises a question of whether similar effects could be observed if, in the absence of any overt behavior, different faces are just passively viewed (i.e. processes such as maintenance of perceptual information in working memory, decision-making and response planning/execution are not involved).

In the current study, we used the steady-state visual evoked potential (SSVEP)^10–12^ approach to investigate the processing of present self-face and past self-face (i.e. the self-face in adolescence and early adulthood). Briefly, the SSVEP is an oscillatory/repetitive response to periodically presented stimuli in which the frequency of the electrocortical response recorded from the scalp mirrors the driving frequency^10^. A critical factor for the definition of a response as an SSVEP is the fact that the stimulation and the response are both periodic^13^. The major difference between the ERP and SSVEP is that ERPs are responses to individual stimuli, whereas SSVEPs are responses to the whole stimulation period. SSVEPs are less prone to ocular artifacts^14^ and electromyographic noise contamination^15^. As noted by Retter and Rossion^12^, SSVEPs are a well-suited approach for studying the discrimination of faces at a neural level. This technique is not only characterized by a high signal-to-noise ratio (SNR) but also offers many advantages like recording speed, objective identification, and quantification of the response of interest^11^. It may be also more sensitive to the activity of neurons that encode different types of faces than other neuroimaging and electrophysiological techniques^11^. Because of this characteristic, the SSVEP approach has often been used in recent studies on face processing^10–12,16^. Taking into account our research question, another important advantage of this approach is that SSVEPs can be measured in the absence of an overt behavior (i.e. with no behavioral task) and thus are not influenced by decision-criterion effects occurring after the sensory or perceptual encoding stages^13^. Therefore, SSVEPs seemed to be well-suited to investigate the impact of the temporal perspective on self-face processing.

First of all, we tested whether SSVEP responses are indeed face-specific. Such face-specificity (i.e. higher amplitudes of SSVEP to faces than other stimuli) has recently been reported when briefly presented images of variable faces were compared to images of body parts or houses presented under the same stimulation conditions^17^. In that SSVEP study, images of faces, body parts and houses were intermixed with stimuli that belonged to different categories (e.g. furniture, birds). In the present study, a different approach was used to further investigate the face-specificity of periodic brain responses. So-called scrambled faces were included in a set of stimuli. Importantly, faces and scrambled faces were presented in separate stimulation sequences.

In order to control for the impact of familiarity *per se* on SSVEP responses, close-other’s and unknown faces were introduced as control stimuli to present and past self-faces that are highly familiar to oneself. Similarly to other studies^8^, the close-other (a person who is the most significant ‘at present’, i.e. at the time of our experiment) was freely chosen by each participant. By this, we wanted to avoid a situation in which a pre-defined person (e.g. father) is not really close to a particular subject. Since Butler *et al*.^9^ proposed the exposure factor as one of the major agents influencing the neural correlates of self- vs. other face processing, the duration of relationship with the close-other was controlled.

Previous SSVEP studies on face processing demonstrated that repetitions of the same face results in reduced SSVEP when compared with the presentation of different faces, suggesting high-level neural adaptation to repeated stimulus^10–12^. Following this approach, identical-faces and different-faces stimulations were introduced in the current study. This is because we were interested whether all types of stimuli (present self, past self, close-other, unknown, scrambled) would result in similar neural adaptation. Importantly, in earlier studies the ‘identical faces’ stimulation consisted of one face image of the same person, being presented many times and the ‘different faces’ stimulation consisted of face images of different people. In the present study, however, identical-faces and different-faces conditions were defined differently. Namely, in the identical-faces conditions the *same* image of the present self-face, the past self-face, the close-other’s face, and face of an unknown person’s was used. In the different-faces conditions, *different* images of the present self-face, the past self-face, the close-other’s face, and the face of an unknown person served as stimuli. In order to enable direct comparison of faces vs. scrambled faces, similarly to faces, scrambled faces were presented in analogous ‘identical’ and ‘different’ conditions, i.e. either one scrambled face or different scrambled faces flickered in a given stimulation session.

Finally, we were interested whether the self-preference effect in face processing, commonly observed in fMRI and ERP studies^8,18–21^ would emerge in our SSVEP investigation. This effect seems not to be influenced by attentional manipulations and task-relevance, indicating an automatic process of self-face recognition/identification^19,22^.

Taking into account the findings of the aforementioned studies, in the present study we took advantage of the human brain’s precise synchronization to periodic input in order to verify the following hypotheses: (i) SSVEP to faces will be enhanced in comparison to SSVEP to scrambled faces; (ii) stimulation with different faces will result in enhanced SSVEP in comparison to stimulation with identical faces; (iii) SSVEP will show the self-preference effect and the highest responses will be observed for stimulation with self-faces, especially present ones; (iv) the absence of top-down processes will reveal differences between the present and past selves, therefore resulting in different SSVEP responses.

In addition, we investigated the sources of SSVEP responses to faces. The involvement of the fusiform gyrus in face processing is well-documented in numerous fMRI studies^23,24^. Sequential presentation of face images from different individuals produces higher activation in the fusiform face area, compared to the repetition of faces of the same person^25,26^. This finding has been interpreted as a release from the adaptation produced by repetition of the same face. This resembles the effects observed in SSVEP studies on face processing. Thus it seems reasonable to link face-evoked steady-state potentials to generators within this region^10^. Therefore, we tested to what extent sources located in the fusiform gyrus would explain the recorded activity.

## Results

### Faces vs. Scrambled faces

**Figure 1:**
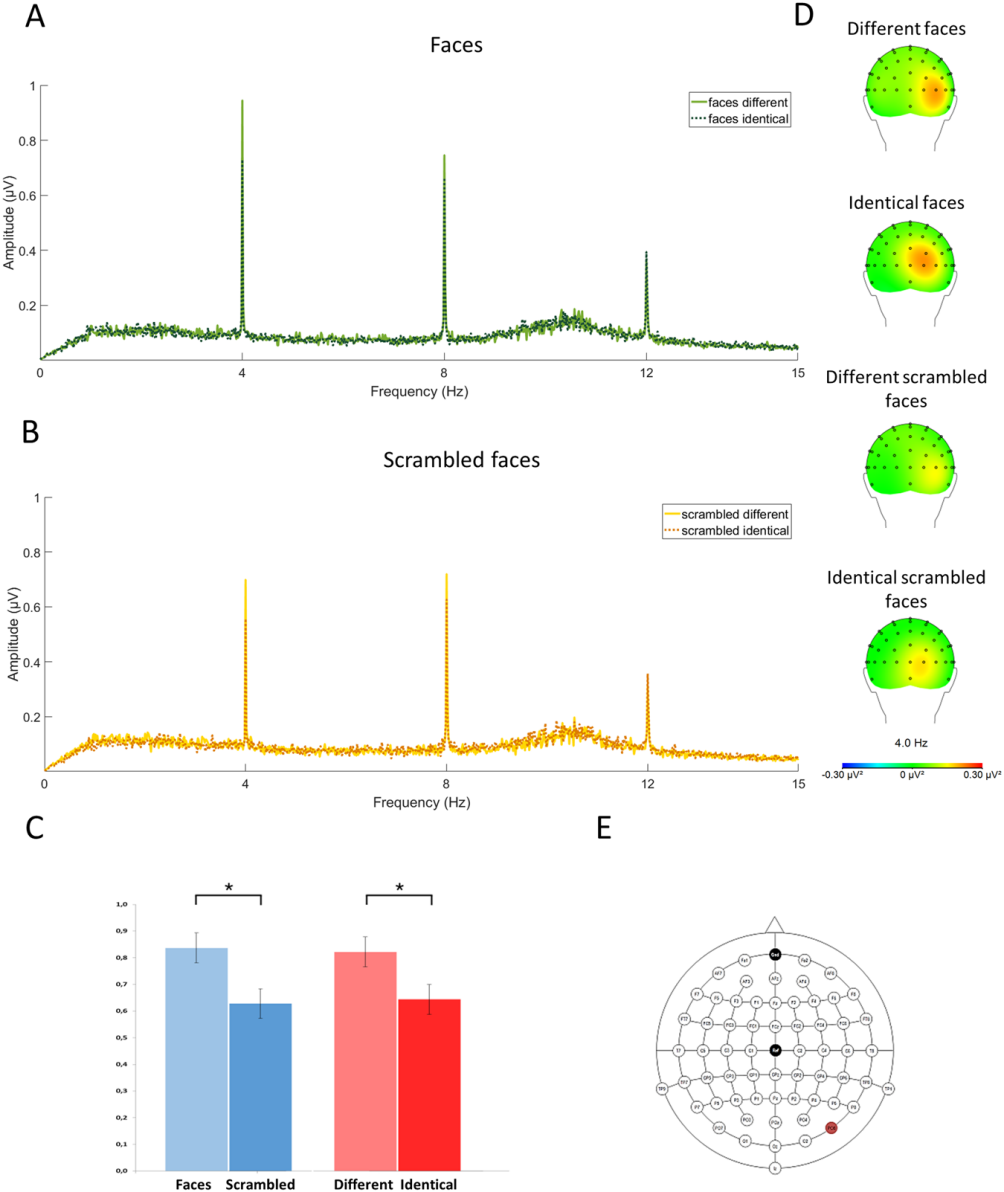
EEG spectrum (at PO8) together with topographical maps of evoked activity at 4 Hz (i.e. frequency of visual stimulation). EEG spectrum for two types of stimulation (different, identical) for faces (**A**) and scrambled faces (**B**). Response at 4 Hz for two classes of stimuli (faces, scrambled faces) and two types of stimulation (different, identical). Asterisks (*) indicate statistically significant differences (*P* < 0.05) (**C**). Topographical maps of evoked power at 4 Hz for each experimental condition (**D**). Extended 10–20 system of electrodes positioning with PO8 marked in red (**E**).

ANOVA yielded significant effects for both main factors: ‘stimulation’ (different, identical; *F*_1,19_ = 8.195; *P* = 0.010; ${\eta }_{p}^{2}$ = 0.301) and ‘stimuli’ (faces vs. scrambled faces; *F*_1,19_ = 5.013; *P* = 0.037; ${\eta }_{p}^{2}$ = 0.209). The interaction of these two was non-significant (*F*_1,19_ = 0.362; *P* = 0.56; ${\eta }_{p}^{2}$ = 0.019). Significant main effects indicated that SSVEP responses to different stimuli were higher than to identical stimuli (0.822 µV and 0.644 µV, respectively) and SSVEP responses to faces were higher in comparison to SSVEP to scrambled-faces (0.837 µV and 0.628 µV, respectively). Figure 1 illustrates those results.

### Different types of faces (present self, past self, close-other’s, unknown)

First of all, the impact of the exposure factor on SSVEP responses to the close-other’s faces was investigated in the following way. The median for the relationship length was calculated, and the group was split below and above 5.5 years into a ‘short relationship group’ (less than 5.5 years, 10 subjects, *M* = 3.95, *SD* = 1.64) and a ‘long relationship group’ (5.5 or more years, 10 subjects, *M* = 16.60, *SD* = 4.74). For the close-other conditions, SSVEP amplitudes in ‘short relationship’ and ‘long relationship’ groups were directly compared using t-test. T-test indicated non-significant results: (*t*(18) = 0.045, *P* = 0.965). The figures illustrating SSVEP responses to the close-other’s face in short and long relationship group can be found in the Supplementary Information (Supplementary Fig. S1). In addition, we correlated amplitudes of SSVEP to close-other’s faces with the duration of relationship with the close-other. This again yielded non-significant results (*r*_*p*_ = 0.12; *P* = 0.61). The effect of the close-other type was also examined. Participants were divided in 3 subgroups based on the fact who had been chosen as the close-other: partner, family member, friend. The length of relationship substantially differed in these subgroups (see description of Supplementary Fig. S2). However, the number of cases in each subgroup was insufficient to run any statistical test (9, 6, and 5, respectively). Therefore, in the main analysis all aforementioned subgroups were collapsed together.

**Figure 2:**
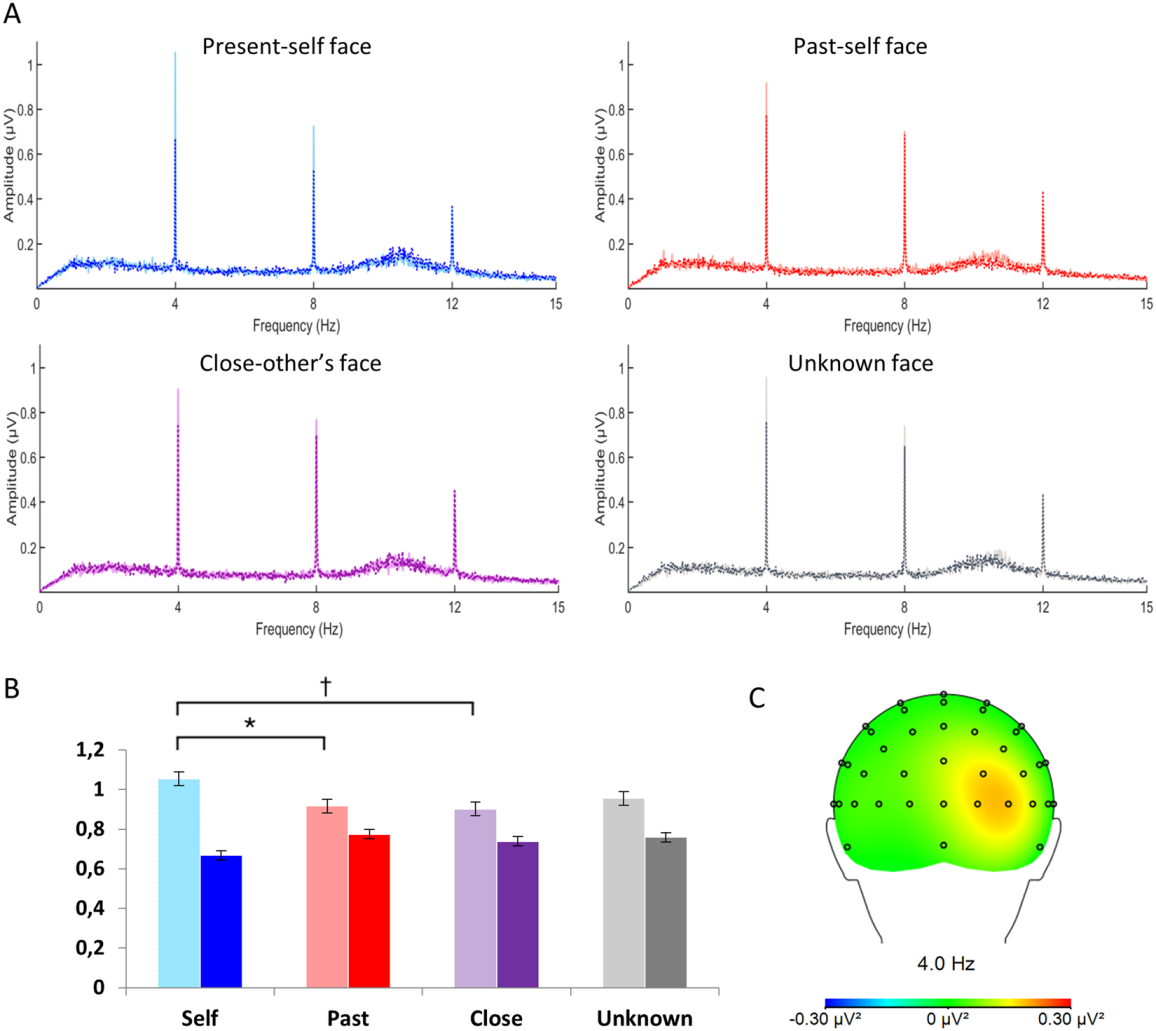
EEG spectra (at PO8) for each type of face: present self, past self, close-other’s, and unknown; different and identical conditions are superimposed (**A**). Maxima at 4 Hz. Bright colors – different faces, dark colors – identical faces. Symbols * and † indicate significant differences (*P* < 0.05) and differences at a trend level (*P* < 0.1), respectively. (**B**). Topographic maps of evoked activity (at 4 Hz) collapsed across all experimental conditions (**C**).

The main analysis taking into account the ‘stimulation’ (different, identical) and ‘type of face’ (present self, past self, close-other’s, unknown) yielded the former factor as significant (*F*_1,19_ = 4.581; *P* = 0.046; ${\eta }_{p}^{2}$ = 0.194), as well as the interaction of the two (*F*_3,57_ = 4.635; *P* = 0.009; ${\eta }_{p}^{2}$ = 0.196). The main factor of ‘type of face’ did not reach the level of statistical significance (*F*_3,57_ = 0.434; *P* > 0.729; ${\eta }_{p}^{2}$ = 0.022). SSVEP responses to different faces were higher than SSVEP responses to identical faces (0.957 µV and 0.735 µV, respectively). *Post-hoc* tests of the significant interaction showed that among different faces, SSVEP to present self-faces was enhanced in comparison to SSVEP to past self-faces (1.054 µV vs. 0.917 µV; *P* = 0.036). SSVEP to present self-faces was also enhanced in comparison to close-other faces but only at a trend level (1.054 µV vs. 0.902 µV; *P* = 0.053). No differences were found between present self- and unknown faces, past self- and close-other’s faces, past self- and unknown faces, as well as close-other’s and unknown faces (all *P*s > 0.99). All comparisons within the identical condition were non-significant (most *P*s > 0.99, present-self vs. past-self *P* = 0.126). Figure 2 illustrates all of those results.

### Source analysis

**Figure 3:**
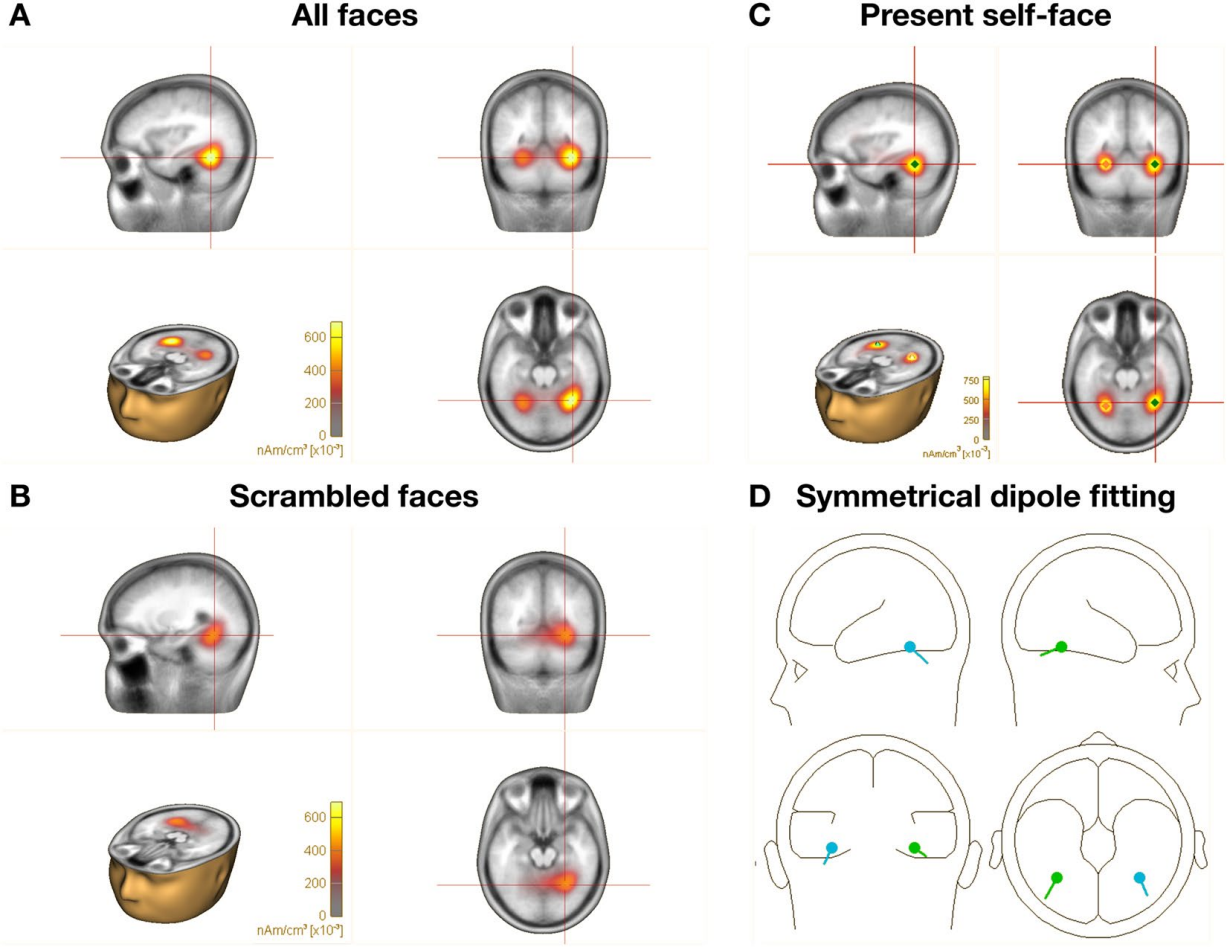
Source analysis of SSVEP responses. Distributed source imaging with CLARA (Classical LORETA Analysis Recursively Applied) points to the fusiform gyrus as the most active generator of the signal elicited by presentation of faces (**A**) and scrambled faces (**B**). Results of CLARA imaging for present self-faces (**C**). Two dipoles fitted within the fusiform gyrus explains over 98% of the data (**D**).

Following the pattern of SSVEP results, a source analysis was performed for images of faces combined across all types in order to reveal the general differences between faces and scrambled faces. In the first step, Brain Electromagnetic Source Analysis (BESA®) with use of CLARA source estimation (Classical LORETA Analysis Recursively Applied) revealed the strongest activity in response to faces at coordinates: X = 34.0; Y = −52.4; Z = −18.6, identified by the Tailarach Client 2.4.3 as the fusiform gyrus within +/−5 mm cube range around the peak of activation. These coordinates were consistent across all types of faces. The coordinates for scrambled faces, however, seemed to be moved slightly anterior, resulting in the maximal signal at coordinates: X = 26.0; Y = −56.4; Z = −14.6 (Fig. 3). The identified dispersed sources successfully explain 98.487% of the data. Moreover, the symmetrical dipole fitting model applied for all the faces explains 98.337% of the data. Both methods provide a good model of the source signal in the fusiform gyrus (Fig. 3). Statistical comparisons of present self-faces vs. scrambled faces can be found in Supplementary Information (Table S1). Supplementary Figure S3 shows results of CLARA source estimation for each type of face (present self, past self, close-other, unknown).

## Discussion

The concept of self is mainly investigated through self-related stimuli such as one’s own face, name or body^18,27^. However, the self includes countless related aspects regarding, among others, continuity across time^3,4^. In the present study, we aimed to investigate time-related changes to the physical aspects of the self, conceptualized as age-related changes of the self-face. We used a modified version of fast periodic visual stimulation (FPVS) to evoke steady-state visual potentials, often used in the current research on face processing^10–12,16^. The modification referred to the type of faces used within a given session of stimulation, i.e. the present and past self-face, close-other’s face, and unknown face as either one repeatedly presented image (‘identical’ stimulation) or different images of the same face type (‘different’ stimulation).

In general, the findings of the present study supported our predictions. First of all, we observed the face-specificity of SSVEP: in comparison to scrambled faces, faces evoked higher SSVEP. This finding is in line with Jacques *et al*.’s recent study^17^ that reported a similar effect for faces vs. other complex visual stimuli (houses, body parts). Moreover, we observed a similar impact of the ‘identical’/‘different’ stimulation on faces and scrambled faces: in both cases, ‘different’ stimulations yielded significantly enhanced SSVEP when compared to the ‘identical’ stimulations.

Higher brain responses to different faces than to identical faces were repeatedly reported in SSVEP research on face processing^10–12,16^. Decreased neural activity to identical faces has been linked to the well-known phenomenon of neural adaptation, also termed repetition suppression or habituation; it refers to the attenuation/reduction of neural activity as a result of immediate repetition of the same stimulus^10,28–30^. Such an effect was initially observed in monkey studies in which so-called stimulus specific adaptation of single unit activity was found^31,32^. Briefly, for many cells in the ventral temporal lobe of macaque monkeys, previous exposure to a visual image reduced the amplitude of subsequent responses to the experienced image without reducing the responses to novel images, i.e. this reduction in response was specific to the re-presentation of the original stimulus^32^.

Importantly, in the different-faces condition of SSVEP studies a stimulus (face) is not re-presented in a consecutive trial, thus implying a lack of neural adaptation^10^. Increased neural activity to different faces presented within one stimulation sequence have been typically viewed as a consequence of individual face identity discrimination^10–12,16^. However, we observed a similar effect for scrambled faces that have no ‘identity.’ In line with numerous monkey^31,32^ and fMRI studies^28–30^ that used visual stimuli other than faces, increased SSVEP both to different faces and different scrambled faces seems to be related to a lack of neural adaptation to *any* visual stimulus in general, and may be associated with the process of change detection in a series of visual inputs.

Differences between SSVEPs to specific types of faces (the present self, the past self, the close-other, and an unknown person) were found only for the ‘different faces’ stimulation. SSVEPs in the ‘identical face’ stimulation were not influenced by type of faces, thus suggesting that neural adaptation was similar in all cases. Specifically, for the ‘different faces’ stimulation, the present self-faces evoked a significantly higher SSVEP responses than past self-faces and a similar trend was found for present self- vs. close-other’s faces. Thus, the present study provided evidence for neural differentiation of the physical aspects of the present and past selves when faces were just passively viewed by participants, without any behavioral task to be performed. In contrast, previous ERP studies^8,9^ that reported similar brain responses to present and past self-faces required an overt behavior that was based on top-down, goal-oriented processes: either face identification^9^ or face detection^8^. Therefore, differences between those ERP findings and the results of the current SSVEP study may be attributed to different involvement of top-down processes, present in the former and absent in the latter.

Moreover, we observed that SSVEP responses to present self-faces were higher than to close-other’s faces, but only at a trend level. The issue of similarities/dissimilarities between the neural correlates of the (present) self and the close-other is a matter of ongoing debate and more experimental evidence is required to resolve this issue. Nevertheless, this finding is in line with previous ERP studies, showing significant neural differences between the processing of the self- and the close-other’s faces^8,19^.

It should be noted that similarly to previous SSVEP studies on face processing^10–12,16^, our SSVEP analyses were done for EEG data recorded within the right parieto-occipito-temporal region that was identified as a region of maximal activity (based on topographical maps of evoked power collapsed across all experimental conditions). Discrete source localization clearly indicated that generators in the fusiform gyrus explained 98% of the data for all types of faces collapsed together. In addition, the slightly different locations of neural generators for faces and scrambled faces are in line with evidence indicating separate generators for faces and objects, yet both are still located in the fusiform gyrus^33–35^. It is worth noting that discrete source analysis as well as SSVEP showed a similar pattern of activations indicating higher response to faces in comparison to scrambled faces (see Figs 1 and 3).

Future SSVEP studies on present and past self-face processing may take into account our results and the recent findings of Jacques *et al*. and Rossion *et al*.^17,36^. In Jacques *et al*.’s and Rossion *et al*.’s studies a novel paradigm was applied to identify an objective signature of face categorization. Images of objects were presented at a frequency of 5.88 images/s whereas images of faces were interleaved every five stimuli, i.e. at 1.18 Hz. In these FPVS sequences, common low- and high-level visual processes between faces and other objects were captured at the 5.88 Hz frequency, while high-level category-selective responses (related to face categorization) were quantified at the 1.18 Hz frequency. We propose that future studies may apply this paradigm, with faces of different types (including present and past self-face) presented at a given fixed frequency, intermixed with other stimuli presented at some basic frequency. In this way, such studies will use all the advantages of the SSVEP approach, and the results will provide a strong evidence for the role of face type on SSVEP responses.

In conclusion, the results of this study showed the face-sensitivity of SSVEP. Crucially, SSVEP to the present and past self-faces differed, indicating that physical aspects of the present and past self are clearly differentiated on the neural level. These effects were evident in the absence of an overt behavior.

## Materials and Methods

### Ethic statement

The research protocol was approved by the local Ethics Committee (SWPS University of Social Sciences and Humanities, Warsaw, Poland) and all methods were performed in accordance with the relevant guidelines and regulations. Informed consent was obtained from all subjects. All subjects received financial compensation for their participation in the study.

### Participants

20 healthy adult participants with normal or corrected-to-normal vision were recruited for the study (10 females). The mean age of participants was 25.8 (*SD* = 2.5). No subjects were excluded from the study and none reported any neural or psychological disorders. Participants’ handedness was checked with the adapted Edinburgh Inventory^37^. All of the participants were right-handed.

### Stimuli

Images of faces were presented to the participants in sets referring to the particular type of face (present self, past self, close-other, unknown). Photographs were collected from the participants and the ‘present self’ set contained the ten most recent photos taken maximally six months prior to the study. The ‘past-self’ set included ten photographs taken at the time of adolescence. The ‘close-other’ set contained ten pictures of a declared closely related person (friend/partner/family member etc.) taken recently, i.e. maximally six months prior to the study.

Similarly to our previous studies instead of a pre-defined close-other we decided to ask participants to freely choose a person with whom they felt strongly emotionally connected to at the time of the experiment^8,19,21,38–40^. Among all the participants, nine subjects chose their partner, six – a family member, and five – a friend as the closest person. The mean duration of relationship was 10.3 years (*SD* = 8.36).

The ‘unknown face’ set consisted of ten photos of a person not familiar to the subject. With the permission from our participants, images of their faces were used as unknown stimuli for other subjects they were unfamiliar with. Furthermore, the ‘scrambled face’ set consisted of ten pictures taken from A series of the Karolinska Directed Emotional Faces database^41^, in which the classical shape of the face was disturbed.

All pictures were rendered grayscale and displayed on a black background. Faces were extracted from their backgrounds and put into a oval, rendering only the face visible with usage of Adobe Photoshop CS5®. Picture luminance was matched to the color statistics of a single picture using Adobe Lightroom CC®. All pictures were resized to the height of 198 pixels, resulting in a visual angle of 3° × 5°.

### Procedure

After electrode cap placement (ActiCAP, Brain Products, Munich, Germany), participants were seated in a light- and sound-attenuated room, at a viewing distance of 60 cm from the computer screen (Eizo Flex Scan EV-2450, Hakusan, Ishikawa, Japan). The screen was specially calibrated for correction to black in order to avoid exhausting the eye with intense background illumination. The experimental procedure was designed using Presentation® software (Neurobehavioral Systems, Albany, CA, USA).

Fast periodic visual stimulation (FPVS) was applied. The frequency of stimulation was kept constant along the entire experiment at a rate of 4 images per second (4 Hz). This low stimulation rate allows to record SSVEPs generated by higher level visual processes, such as the discrimination of complex facial stimuli^13^. Moreover, in the context of source analysis, it was crucial to obtain segments of signal of sufficient length between the stimuli occurrence. A higher rate of stimulation, e.g. the 6 Hz suggested by Alonso-Prieto *et al*.^42^ as an optimal frequency for face discrimination, would not allow for an appropriate length of extracted segments. In order to model the sources of signal around 170 ms^43^, it was reasonable to obtain at least 220 ms of the signal, which captures both the rise and fall of the N170 component. For this reason, the 4 Hz frequency was chosen in our experimental design.

The stimulation was given as follows: in each sequence of stimulation a face (or scrambled face) appeared and disappeared on the screen with a rate of stimulation of 4 faces/s (one face every 250 ms). The frame rate of the LCD monitor was set at 60 fps (frames per second), resulting in one frame being visible for around 16.7 ms. Each face was visible for a little less than half of a cycle (117 ms), being displayed over 7 frames.

We decided to opt for a square wave stimulation. Importantly, it was proven that either sinusoidal or square shaped visual stimulation generates harmonics as a consequence of the nonlinear properties of neural populations^13,44^.

Five types of stimuli (present-self, past-self, close-other, unknown, and scrambled) were presented in two versions: one picture being presented for the entire trial (the *identical* condition), or ten different pictures presented in a pseudo-randomized order such that no picture followed itself (the *different* condition). It is important to note that in the *different* condition ten different pictures of the same person were displayed.

The experimental session was divided into three runs. Each run consisted of 10 different experimental conditions (5 types of faces × 2 types of stimulation), with 10 minutes breaks in between. The stimulation sequence in each experimental condition lasted 90 seconds, with a 15 seconds break between runs. Each occurrence of a stimulus was marked by a trigger sent to the EEG recording device. The experimental conditions were randomly assigned to the stimulation sequences in each run. Each run lasted around 16 minutes and a break was scheduled to separate the sessions. The total length of experiment was about 70 minutes.

In order to compare the face vs. non-face stimuli, scrambled images were compared to pseudo-randomly selected faces. There were only 6 trials with scrambled faces (3 identical and 3 different), but 24 trials with normal faces: 4 types of faces (present-self, past-self, close-other, and unknown) × 3 runs × 2 types of stimulation (identical or different). In order to avoid overbalancing of faces, a pseudo-random draw of 6 face trials (3 identical and 3 different) was performed, creating a set of face trials equal in number to the scrambled stimuli trials and unbiased in respect to the type of face. With this solution different conditions were equally represented in the group of faces. Fast Fourier Transform (FFT) was calculated for the drawn conditions, averaged across 3 runs, and amplitudes spectra were analysed.

### EEG recording

EEG was continuously recorded from 64 scalp electrodes using a 128-channel amplifier (Quick Amp, Brain Products, Enschede, Netherlands) and BrainVisionRecorder® software (Brain Products, Gilching, Germany). Ag-AgCl electrically shielded electrodes were mounted on an elastic cap (ActiCAP, Munich, Germany) and positioned according to the extended 10–20 system. Electrode impedance was kept below 5 kΩ. The EEG signal was recorded against an average of all channels calculated by the amplifier hardware. The sampling rate was 1000 Hz.

### EEG analysis

Off-line analysis of the EEG data was performed using custom scripts running on Matlab R2016b (MathWorks, USA), Brain Vision Analyzer 2.0® (Brain Products GmbH, Munich, Germany) and BESA Research 6.1 (BESA GmbH, Grafelfing, Germany).

#### Preprocessing

First, a Butterworth zero-phase filter was applied with the frequency band from 1 to 30 Hz and order 1. Then the data were re-referenced to the mean signal recorded from the earlobes. The continuous signal was segmented into 90 s blocks of stimulation according to the conditions markers. The initial 30 s of the signal was removed from the data in order to avoid the effects of SSVEP rising or fast neural adaptation^10^. The re-segmenting was performed precisely in reference to the 120^th^ stimulus in the sequence. The timing of the flicker with respect to trial onset was the same in every trial. The trials from three separate runs were then averaged within each condition. FFT was applied to the remaining 60-s long averaged segments of stimulation, giving a very high frequency resolution (1000/60000 = 0.017 Hz). Thus, the amplitude spectrum (µV) at the frequency of interest 4 Hz was extracted for each condition separately taking the maximal value between 3.97–4.03 Hz.

Selection of electrodes for analyses has to be orthogonal to potential differences between experimental conditions^45^. Thus, such as selection has to be done on the basis of topographical distribution of brain activity averaged across all experimental conditions^46^. Electrodes within maxima identified in such a topographical map should be further analyzed. Based on topographical distribution, the grand averaged amplitude spectra were generated for the right parieto-occipito-temporal site, PO8, which lies in accordance with previous studies^10^.

#### Statistical analysis

In order to compare faces vs. scrambled faces statistical comparisons were performed using two-way repeated measures ANOVA with factors of ‘stimulation’ (different vs. identical picture) and ‘stimuli’ (faces vs. scrambled faces).

Following the faces vs. scrambled faces analysis, a two-way repeated measures ANOVA was performed on amplitude spectra with ‘type of face’ (present-self, past-self, close-other, unknown) and ‘stimulation’ (different vs. identical) as factors.

All effects with more than one degree of freedom in the numerator were adjusted for violations of sphericity^47^. Bonferroni correction for multiple comparisons was applied to the post-hoc analyses. The analyses were conducted in IBM SPSS Statistics 21 Advanced Model.

### Estimation of sources

To model the sources of the signal, similar steps of preprocessing were applied to the data as described above. Source estimations were based on a total of 2160 trials representing a given type of face across the entire experiment. Segments 200 ms-long after the stimulus onset were extracted from the data and averaged over the subjects. A clear negative component was observed, peaking at a latency of 142 ms. Only the rising of the component (119–144 ms) was taken into source estimation, as it resembles the actual neural postsynaptic activity^48^. The source analysis was performed with BESA Research 6.1 and Analyzer 2.0 software. Two methods of source analysis were applied: discrete source analysis (dipole fitting) and two alternative distributed source imaging methods, LORETA (Low Resolution Electromagnetic Topography) and CLARA (Classical LORETA Analysis Recursively Applied), both implemented in BESA Research 6.1.

#### Discrete source analysis

On the basis of neuroimaging research, the fusiform gyrus is claimed to be one of the most important structures involved in face recognition^49–51^. Therefore two dipoles were placed symmetrically in the area of the fusiform gyrus in the left and right hemisphere and fitted automatically in order to find the best model explaining our data. The dipole fit is calculated in several repeated steps. First, using the head model, the forward model topography is estimated. Then, the inverse of the estimated topography is applied to the data in order to model the source waveform. This waveform is projected back to the scalp using the forward coefficients of the map to estimate the model signals. The measured and modeled data are subtracted to estimate the residual waves. The model with the least residual value is presented in Fig. 3.

#### Distributed source analysis

Both discrete and distributed source models use dipoles as their basic elements to model brain activity. However, distributed methods use relatively more sensors to model the data. LORETA^52^ and CLARA were used to identify sources and verify the hypothesis underlying the abovementioned discrete model. CLARA, the Classical LORETA Analysis Recursively Applied is an iterative application of the LORETA algorithm with an implicit reduction of the source space in each iteration (BESA 6.1 tutorial^53^).

### Data Availability

The data that support the findings of this study are available from the corresponding author upon reasonable request.

## Electronic supplementary material

Supplementary information
