# Supplementary material for: Present and past selves: a steady-state visual evoked potentials approach to self-face processing
Source: Sci Rep. 2017 Nov 27;7:16438. doi: 10.1038/s41598-017-16679-6 (PMC5703895; doi:10.1038/s41598-017-16679-6)
Supplement: Supplementary file 1 — Supplementary information [file 41598_2017_16679_MOESM1_ESM.pdf]

SUPPLEMENTARY INFORMATION

Present and past selves: a steady-state visual evoked potentials approach to self-face processing

Kotlewska I, Wójcik MJ, Nowicka MM, Marczak K, Nowicka A

Supplementary Table S1. Results of present self-faces vs. scrambled faces comparisons for 10 locations within the fusiform gyrus.

						Present self-face vs. scrambled faces	
	**Regions of Interest**	**Side**	***x***	***y***	***z***	***t* score**	***P* (corrected)**
1	Right Fusiform Gyrus BA37	R	40	-47	-15	2.781	.012
2	Fusiform Gyrus	R	44	-40	-20	2.899	.009
3	Right Fusiform Gyrus BA19	R	39	-65	-10	1.768	.093
4	Right Fusiform Gyrus BA19	R	34	-73	-10	1.307	.207
5	Middle Fusiform Gyrus (rFFA)	R	38	-44	-28	2.574	.019
6	Posterior Fusiform Gyrus (rOFA)	R	44	-76	-14	1.714	.103
7	Right Fusiform Gyrus	R	38	-55	-20	2.673	.015
8	Right Fusiform Gyrus	R	40	-54	-12	2.151	.045
9	Brodmann area 19 R	R	33	-75	11	1.738	.098
10	Brodmann area 37 R	R	45	-51	-12	2.662	.015

Coordinates (in standard stereotaxic space[1]) refer to maximally activated loci within area of activation.

Several different coordinates were included into present self-faces vs. scrambled faces comparisons. Selection of locations 1, 3, 4 was based on Li et al.'s study[3], 5 and 6 on Rossion et al.'s study[2], and 7 on Sabatinelli et al.'s study[4]. Locations 2, 9, and 10 were taken accordingly to the coordinates implemented in Analyzer 2.0 that correspond to the selected areas. T-tests indicated the significantly stronger activity for present self-faces in six out of ten locations.

Supplementary Figure S1. EEG amplitude spectrum (at PO8) for the ‘different’ type of stimulation. SSVEP to close-other’s faces are plotted separately for ‘short relationship’ and ‘long relationship’ groups.

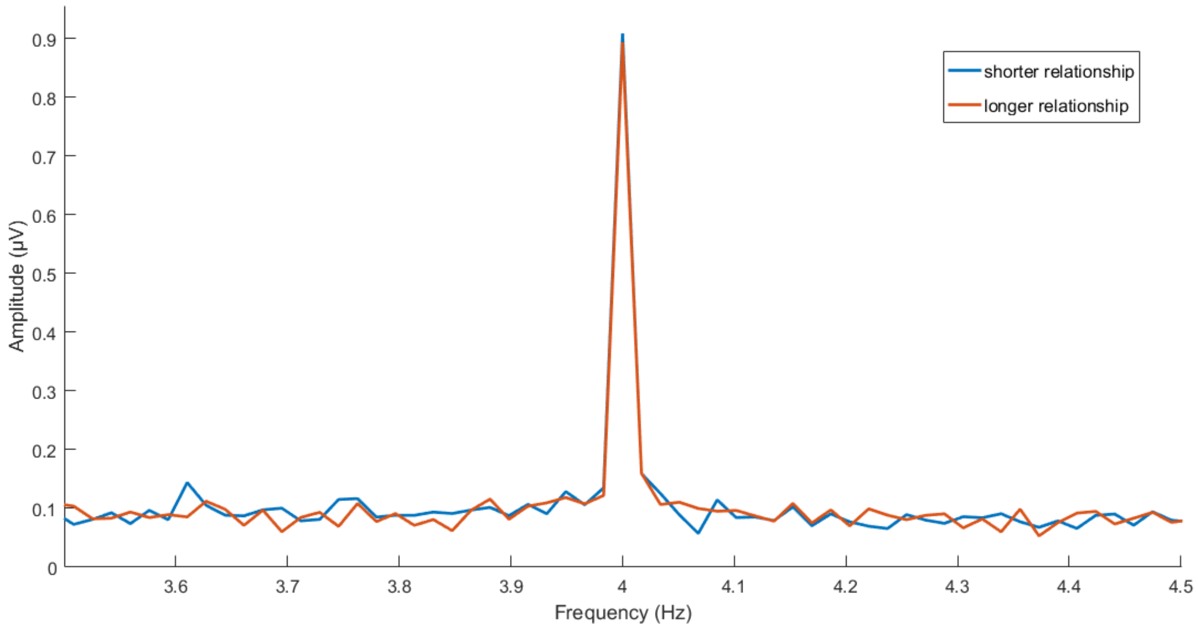

Taking into account the length of relationship with the close-other, the group of participants was subdivided into ‘short relationship group’ and ‘long relationship group’, based on the calculated median (5.5 years). SSVEP amplitudes in the two groups were compared using the t-test. It indicated a non-significant result: ($t(18) = 0.045$, $P = 0.965$). In addition, amplitudes of SSVEP to close-other’s faces were correlated with the duration of relationship with the close-other. This yielded also a non-significant result ($r_p = 0.12$; $P = 0.610$).

Supplementary Figure S2. EEG amplitude spectrum (at PO8) for the 'different' type of stimulation. SSVEP to close-other's faces are plotted separately for three types of the close-other: partner, family member, and friend.

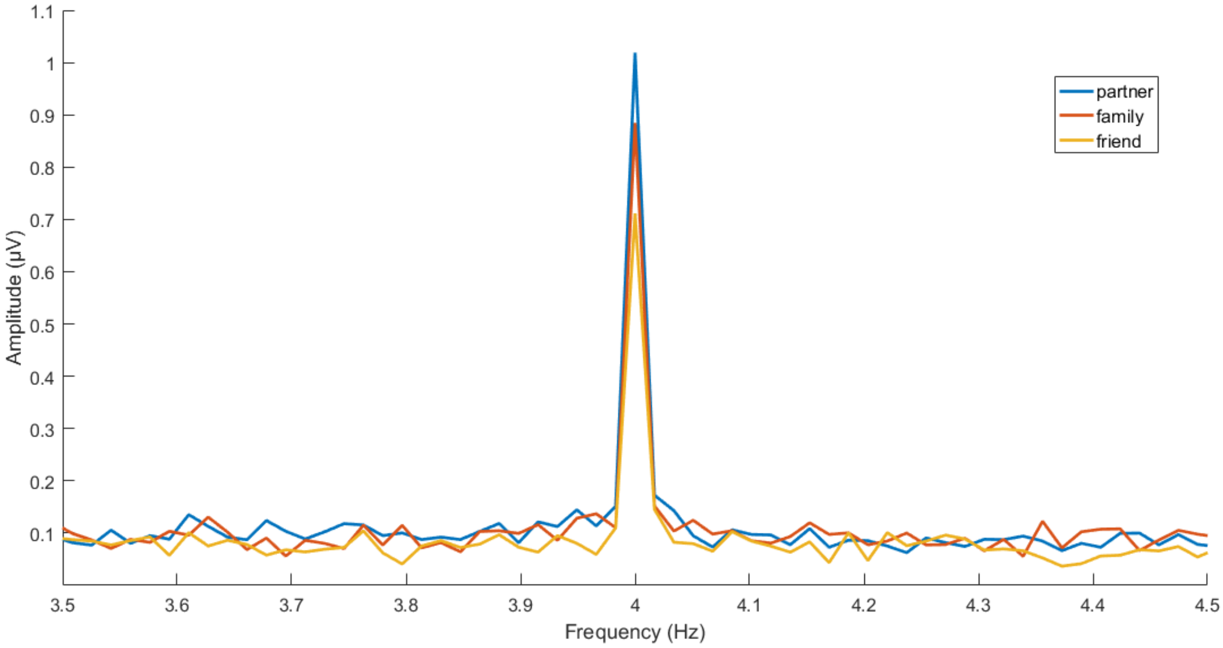

The impact of relationship-length with the close-other on SSVEP responses was approached yet in another way. Participants were divided in 3 subgroups based on the fact who had been chosen as the close-other: 1st group (9 participants) – partner, 2nd group (6 participants) – family member, and 3rd group (5 participants) – friend. The length of relationship substantially differed in these subgroups: 1st group – 4.22 ± 1.99, 2nd group – 20.50 ± 5.82, and 3rd group – 8.9 ± 6.31. We checked amplitudes of SSVEP to close-other's faces in each subgroup and mean values ± SD were as follows: 1st group – 1.018 ± 0.94; 2nd group – 0.88 ±0.57; 3rd group – 0.71 ± 0.48. However, the number of cases in each subgroup was insufficient to run any statistical test. Nevertheless, the mean amplitude of SSVEP to close-other's faces was the highest in the case of partner's faces and the lowest – in the case of friend's faces, with SSVEP responses to family member's faces in between.

Supplementary Figure S3. Source analysis of SSVEP responses. Distributed source imaging with CLARA (Classical LORETA Analysis Recursively Applied) points to the fusiform gyrus as the most active generator of the signal elicited by presentation of each type of face: present self, past self, close-other's, unknown.

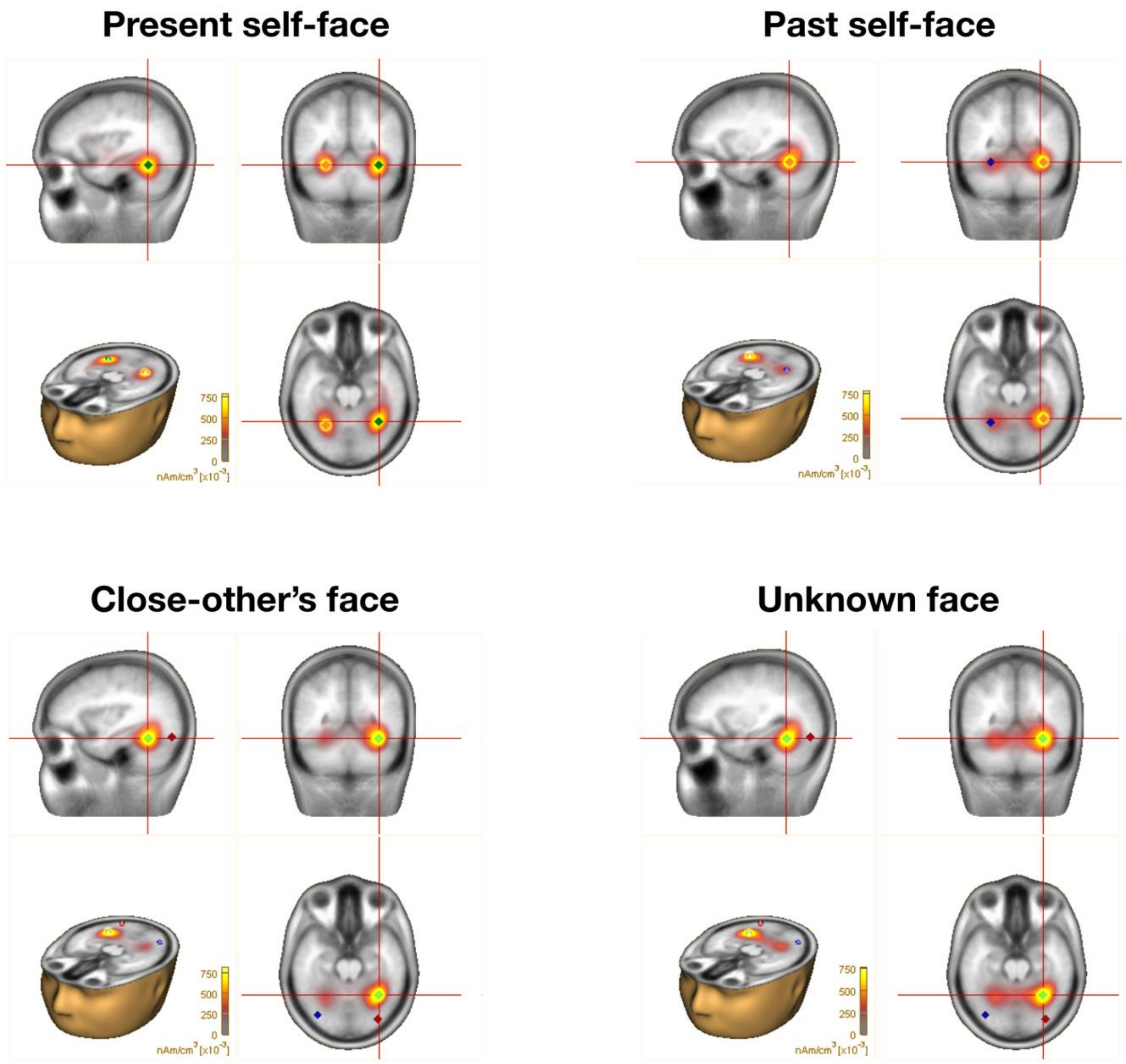

References

1. Talairach, J., & Tournoux P. *Co-planar Stereotaxic Atlas of the Human Brain. 3-dimensional Proportional System: An Approach to Cerebral Imaging*. New York: Thieme Medical Publishers (1988).

2. Rossion, B., Schiltz, C., & Crommelinck, M. The functionally defined right occipital and fusiform "face areas" discriminate novel from visually familiar faces. *Neuroimage* **19,** 877–883 (2003).

3. Li, H., Chan, R.C.K., Mcalonan, G.M., & Gong, Q. Facial emotion processing in schizophrenia: A Meta-analysis of Functional Neuroimaging Data. *Schizophr. Bull.* **36,** 1029–1039 (2010).

4. Sabatinelli, D., et al. Emotional perception: Meta-analyses of face and natural scene processing. *Neuroimage* **54,** 2524–2533 (2011).
